# NAViFluX: a visualization‑centric platform for interactive analysis, refinement and design of genome‑scale metabolic networks

**DOI:** 10.1093/bioinformatics/btag191

**Published:** 2026-04-20

**Authors:** Manjunatha Beduru Krishnamurthy, Pasupula Sridhar Harish, Abhishek Subramanian

**Affiliations:** Department of Biotechnology, Indian Institute of Technology Hyderabad, Telangana 502284, India; Department of Biotechnology, Indian Institute of Technology Hyderabad, Telangana 502284, India; Department of Biotechnology, Indian Institute of Technology Hyderabad, Telangana 502284, India

## Abstract

**Motivation:**

Genome-scale metabolic network (GSMN) models enable flux-based metabolite fate discovery, metabolic engineering, drug target identification, and multi-omics integration. However, programming requirements, architectural complexity, and limited visualization support impede its adoption by the broader scientific community. Existing tools exclusively specialize in GSMN analyses or visualization while lacking important features such as pathway-specific views, database-integrated refinement, and comprehensive enrichment and perturbation analyses.

**Results:**

Here, we present NAViFluX (metabolic Network Analysis and Visualization of Flux), a visualization-centric, web browser-based tool that unifies native pathway/subsystem map generation, interactive model refinement via KEGG/BiGG, pathway merging and modules for flux computations, topology, and functional enrichment all within network views. Using three independent case studies on *Escherichia coli*, the utility of NAViFluX for characterization of nutrient-specific metabolic adaptations, enhancing gene essentiality predictions and interpretability, and rational design of an optimized carbon-fixing metabolic state is demonstrated.

**Availability and Implementation:**

All source code and supplementary files associated with the case studies are publicly available via Zenodo at https://zenodo.org/records/19107831. NAViFluX can be easily installed as a standalone software through https://github.com/bnsb-lab-iith/NAViFluX.

## Graphical Abstract

**Figure:**
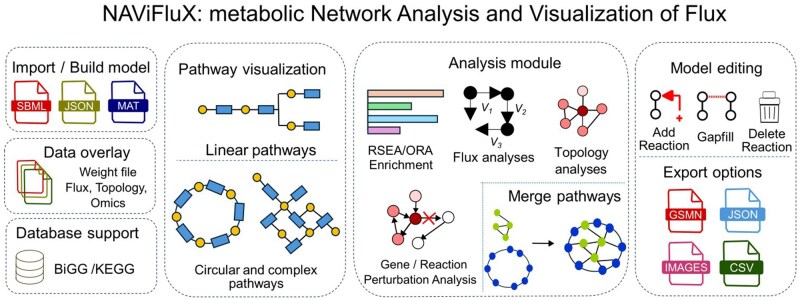

## 1 Introduction

Genome-scale metabolic network models (GSMNs) are comprehensive libraries of gene-associated and non-gene-associated metabolic reactions occurring within an organism (Mendoza et al. 2019). Starting with the annotation of metabolic enzymes encoded within an organism’s genome, GSMNs represent a repository of metabolic reactions rigorously curated for mass and stoichiometry balance, functional characterizations, pathway annotations and cross-database references, a level of curation that even popular resources KEGG (Kanehisa et al. 2025), MetaCyc (Caspi et al. 2020) often lack. Beyond serving as reaction repositories, GSMNs enable flux tracing through metabolic pathways, optimization of cellular metabolism for bioproduction, identification of potential drug targets through *in silico* gene knockout simulations, and integration of multi-omics data to generate condition- or phenotype-specific models (Carter et al. 2023).

Despite their usefulness, routine utility of GSMNs by non-expert biologists remains limited due to the sheer complexity of model architectures (Domenzain et al. 2021). Available reconstruction and analysis software such as the COBRA toolbox, COBRApy, COBREXA, RAVEN toolbox, and CellNetAnalyzer demand specialized programming expertise in MATLAB, Python, or Julia (Klamt et al. 2007, Agren et al. 2013, Ebrahim et al. 2013, Heirendt et al. 2019, Kratochvil et al. 2022). Several GSMN-specific tools attempt to address these issues. Fluxer and CAVE provide flux balance analysis (FBA)-centered exploration, with Fluxer offering flux-oriented network visualizations and CAVE focusing on model editing and global visualization (Hari and Lobo 2020, Mao et al. 2023). ModelExplorer supports structural consistency checks and manual curation but fails to offer intuitive global visualizations and associated analyses (Martyushenko and Almaas 2019). Grohar provides automatic subnetwork visualization and predicts knockout effects on local neighborhood of a given metabolite (Moškon et al. 2018). None of these tools perform topology, functional enrichment, or comprehensive perturbation analyses. MetExplore supports network exploration, refinement, omics-tailored visualizations, and flux analyses, but treats visualization as a post hoc procedure and only supports manual reaction addition without reaction database integration (Cottret et al. 2018). Collectively, existing GSMN analysis tools fail to exploit the complete curated knowledgebase within GSMNs for complementary analyses like pathway enrichment and large-scale gene- or reaction-level perturbations with mechanistic network visualizations.

Alternatively, visualization-centered tools focus on pathway map design from published GSMNs. Escher enables manual construction and editing of pathway maps using pre-drawn template layouts with flux or omics data overlay (King et al. 2015). IMFler loads GSMN flux analyses onto Escher maps (Petrovs et al. 2021). SAMMI provides pathway/subsystem-specific visualizations along with omics overlays, albeit with limited network layouts and no model editing functionalities (Schultz and Akbani 2020). In summary, most GSMN-specific analysis tools emphasize individual workflow steps rather than providing a comprehensive, integrated, visualization-centered analyses workbench. On the other hand, visualization-oriented tools only generate maps, do not support topological/flux analyses and provide limited support towards network refinement. Without native integration of reaction databases, these tools fail to benefit from the prior knowledge to construct metabolic networks. The resultant separation of analyses and visualization also forces format conversions from canonical SBML into tool-specific formats, risking data incompatibility. These limitations underscore the need for a robust, visualization-centric platform capable of modifying GSMNs and performing diverse complementary analyses within a unified framework.

Here, we present NAViFluX (metabolic Network Analysis and Visualization of FluX), a visualization-centric tool with a web-browser user interface that unifies GSMN exploration, refinement, and analysis. NAViFluX provides: (i) native metabolic pathway map generation using annotated GSMN subsystems and intuitive layouts; (ii) interactive model refinement through reaction additions from BiGG/KEGG databases, constraint specification, merging pathways into larger subsystems, and large-scale reaction deletions; (iii) flux analyses modules (FBA, pFBA, FVA, cFBA, gene/reaction knockouts), network topology, and functional enrichment analyses; (iv) overlay of flux, topology metrics, and omics abundances directly onto pathway visualizations; (v) *de novo* metabolic network construction using integrated reaction databases; and (vi) flexible export formats compatible with popular network and flux analysis tools. Unlike existing tools that treat visualization as a post hoc step, NAViFluX positions intuitive network visualization at the center of the workflow, allowing users to make critical decisions directly from what is observed on the network.

NAViFluX was evaluated using three case studies on a publicly available *Escherichia coli* GSMN (Orth et al. 2011, Norsigian et al. 2020), demonstrating its capabilities in characterizing nutrient-specific metabolic adaptations, improving metabolic gene essentiality predictions, providing mechanistic insight into synthetic lethality, and facilitating the rational design of an optimized carbon-fixing metabolic state. Overall, NAViFluX democratizes the exploration of GSMNs for biologically meaningful applications.

## 2 Methods

**Figure 1:**
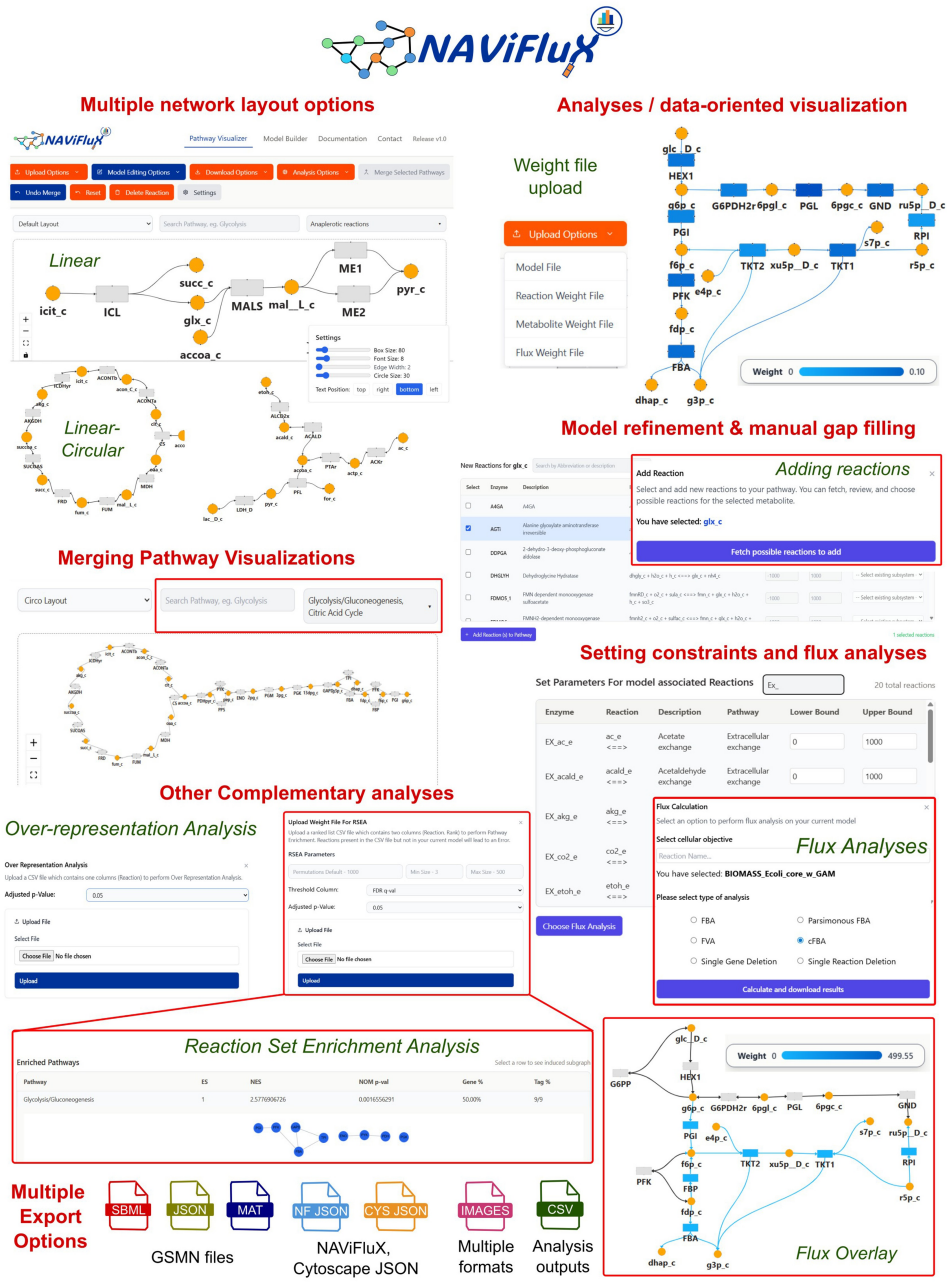
Overview of NAViFluX and its core functionalities.

NAViFluX natively integrates GSMN visualization with model refinement and analyses on-the-fly, supporting an array of input and output file formats. NAViFluX supports two broad modules “Pathway Visualizer” and “Model Builder” whose novel features are highlighted in Fig. 1. Detailed documentation, step-by-step guidance, tutorials, and installation instructions are available within NAViFluX and at https://github.com/bnsb-lab-iith/NAViFluX.

### 2.1 Pathway visualizer module

The “Pathway Visualizer” is the central module of NAViFluX, offering GSMN exploration, visualization, model refinement, and analyses for any uploaded BiGG or KEGG compliant GSMN file (in SBML, MAT, or JSON formats). Upon loading, the parent database (KEGG/BiGG) is automatically detected and the bipartite (reaction-metabolite) pathway-specific networks are generated using the annotated subsystems and the stoichiometric matrix of the GSMN. In each network visualization, metabolites appear as circular nodes, reactions appear as rectangular nodes and the reaction directionality inferred from flux bounds are displayed as reversible or irreversible edges. Currency metabolites are excluded from the visualization to reduce clutter. Network layouts can be manipulated using linear (Hierarchical-LR, Hierarchical-BT), circular (Circo) and hybrid (Stress, Neato, Twopi) visualizations. NAViFluX enables label placement, box/arrow size adjustment, font size, width, adjustment and easy repositioning of nodes and edges within the visualization canvas. Hovering over nodes displays cross-reference IDs (KEGG, BiGG, EC numbers, ChEBI), molecular formulae, and associated weights parsed from the uploaded GSMN.

### 2.2 Model refinement

By identifying the reaction database compatible to the uploaded GSMN format (BiGG/KEGG), users can fetch relevant reaction candidates linked to any selected metabolite from this database, filter by typing reaction abbreviation, name, or description and subsequently update the GSMN. NAViFluX automatically avoids redundant additions. Deletion of unwanted reactions and adaptation of flux bounds and subsystem labels are performed on-the-fly. Also, local gap-filling is supported by selecting two metabolites within the canvas and retrieving connecting reactions from the database. Multiple subsystems can be merged into larger pathways with custom naming, either as a visualization-only merge or as an active GSMN-level merge, with an option to revert.

### 2.3 Flux, topology, and enrichment analyses

NAViFluX implements FBA, parsimonious FBA (pFBA), Flux Variability Analysis (FVA), cycle-free FBA (cFBA), Flux Sampling and single-gene/reaction deletion studies (Tamura 2023) by interactively specifying flux constraints and choosing a cellular objective. Network topology metrics (degree, betweenness, closeness, eigenvector, and PageRank centralities) computed on the bipartite graph are exportable for use in Cytoscape or other tools. Flux and topology metrics are automatically overlaid onto the network and also exported as CSV files. NAViFluX uniquely provides two novel enrichment analyses: Reaction Set Enrichment Analysis (RSEA), a ranking-based approach analogous to GSEA that performs enrichment of GSMN pathway subsystems based on ranked reaction lists and Over-Representation Analysis (ORA) that performs pathway enrichment based on reaction lists provided by the user. Enrichment analyses generate interactive result tables with enriched subnetwork visualizations and CSV exports.

### 2.4 Data overlay

A unique weight-file feature allows overlay of flux measures, topology metrics, omics abundance data or any user-defined measurements as color gradients on reaction/metabolite nodes and edges. Separate weight files are accepted for reactions, metabolites, and flux, with uploaded values standardized for relative comparability. Flux weight files additionally modify edge reversibility arrows to reflect flux direction.

### 2.5 Model builder module

The “Model Builder” module enables metabolic network reconstruction from scratch using BiGG/KEGG databases. Two parallel canvases allow simultaneous construction of subnetworks with independent layout choices, which can then be merged while retaining individual layout arrangements. Models built here can be imported into the Pathway Visualizer for subsequent analyses.

### 2.6 Technical implementation

The NAViFluX build uses a React (v18.2) frontend with Vite, a Flask (v3.1.2) backend communicating via RESTful APIs and ReactFlow (v11.11.4) that handles graph visualization. Backend analyses are implemented in Python (v3.13). COBRApy (v0.30) (Ebrahim et al. 2013) for GSMN parsing and constraint-based optimization, NetworkX (v3.6.1) for topology, and GSEApy (v1.1.11) (Fang et al. 2023) for enrichment analyses were used. GLPK solver was used for performing flux analyses. Network layouts were generated using Dagre (v0.8.5) and ELK.js (v0.10).

### 2.7 Additional analyses for case studies

Nutrient uptake constraints based on M9 and MOPS growth media compositions were inferred using MetaboTools subroutines in COBRA toolbox v3.0 (Aurich et al. 2016). Data preprocessing and statistical analyses were performed in R (v4.4.3) using tidyverse/dplyr, with visualizations generated using ggplot2 and ComplexHeatmap.

## 3 Results

### 3.1 Comprehensive benchmarking of NAViFluX against available GSMN modification, analyses and visualization tools

**Figure 2:**
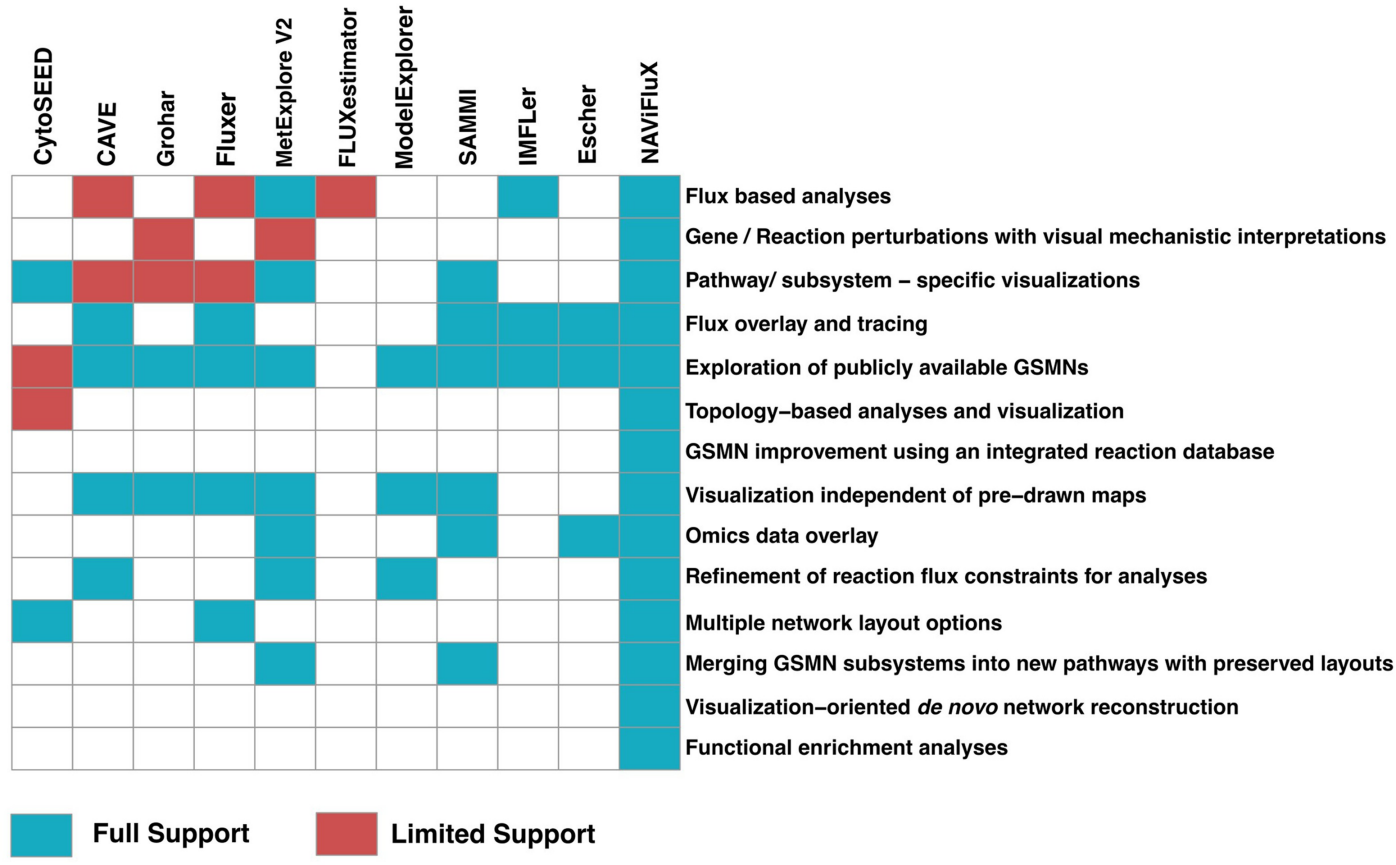
Comparison of NAViFluX features with other GSMN development, analysis and visualization tools. Tools providing full support for certain actions are colored in blue whereas tools providing partial support for those functions are colored in red. The tools are clustered based on overlapping functionalities.

Several GSMN-specific tools assist metabolic network visualization, analyses and refinement, albeit, with significant limitations in scope and flexibility of use. Comparison of NAViFluX features with other tools is provided in Fig. 2.

Analysis-oriented tools like FLUXestimator are purely focused on single-cell flux estimation using human and mouse models with limited visualization support (Zhang et al. 2023). Exclusive model manipulation tools like Model Explorer explore GSMNs and support the refinement of flux constraints (Martyushenko and Almaas 2019). However, model visualizations are global and cumbersome, flux and other functional analyses are absent, no pathway-specific visualization or data overlay options are available. Exclusive visualization-oriented tools SAMMI and CytoSEED offer multiple network layout options with optional subnetwork or pathway visualizations and data overlay (DeJongh et al. 2012, Schultz and Akbani 2020). While SAMMI is restricted to generate force-directed layout visualizations, CytoSEED is restricted to explore only ModelSEED GSMNs (Seaver et al. 2021). Another exclusive visualization tool Escher uses predrawn layouts to generate fully controllable visualizations and supports data overlay (King et al. 2015). IMFLer exclusively performs flux analyses and uses Escher maps for flux overlay with no additional options (Petrovs et al. 2021). FLUXer performs flux balance analyses along with multiple flux-oriented network layout options (Hari and Lobo 2020). Grohar focuses on subnetwork visualization by specifying source and target reactions and can observe the effect of perturbations on local reactions (Moškon et al. 2018). CAVE offers flux-based analyses, subnetwork specific visualization, constraint refinement, force-directed layouts and manual addition of reactions to the GSMNs (Mao et al. 2023). MetExplore V2 performs model building and refinement, analysis, visualization, data overlay and local perturbation/deletion analysis (Cottret et al. 2018). In comparison to all the above tools, NAViFluX provides an integrated workbench that offers novel features for model manipulation, analyses and visualization beyond what is implemented in previous tools. NAViFluX uses an interactive, visualization-centric approach for analysis which allows better visual control of the GSMN. NAViFluX is natively integrated with the BiGG/KEGG database thereby facilitating database-informed expansion of GSMNs (Norsigian et al. 2020, Kanehisa et al. 2025). NAViFluX allows you to iteratively set bounds, perform analyses and immediately observe its consequences helping with decision-making at every step. Another unique feature of NAViFluX is that it cannot only generate pathway-specific visualization but also merge pathway subsystems to generate new pathways with customization options providing flexibility in creating and defining new pathways. It additionally provides an option to merge subsystems actively within the GSMN as well. Using its actively integrated database functionality, NAViFluX can also build models from scratch, a functionality unique to NAViFluX. More importantly, NAViFluX utilizes the curated subsystem definitions of an uploaded GSMN to perform network topology, reaction set enrichment, over-representation and global gene/reaction perturbation analyses. With its integrated workbench approach, NAViFluX can overlay outputs of any of these analyses actively with an independent provision of reaction/metabolite data overlay as well. To summarize, all the different modules of refinement, analyses and visualization can be used cohesively in any combination to make system-level interpretations.

### 3.2 Case studies

Here, we present three case studies to demonstrate the benefits of NAViFluX in performing various analyses and facilitating the interpretation of metabolic mechanisms through network visualizations.

#### 3.2.1 Case study 1: Condition-specific flux analyses and visualizations for the comparison of carbon source utilization by *E. coli*

To demonstrate NAViFluX’s capability for obtaining flux-level insights, we perform an FVA simulation in *E. coli* iJO1366 GSMN and compare it with experimentally detected metabolites outlined in a targeted metabolomics dataset from a previous study (Orth et al. 2011, Radoš et al. 2022). The original study measured 101 primary metabolites across 19 environmental conditions, out of which we used 11 conditions pertaining to only carbon sources for comparison.

Upon uploading the *E. coli* GSMN onto NAViFluX and adapting estimated uptake constraints in the “Flux Analyses” module, FVA was performed with biomass maximization as the objective, to identify “flux-active” reactions (non-zero minimum or maximum rates) in each condition independently (Supplementary File 1); the associated metabolites were termed “flux-active metabolites.” FVA predicted a growth rate of 0.51 mmol/(gDW·hr) for the glucose-only condition, consistent with prior reports (Oftadeh and Hatzimanikatis 2024). Optimal growth rates varied between 0.18 to 0.51 mmol/(gDW·hr) depending upon the carbon source.

**Figure 3:**
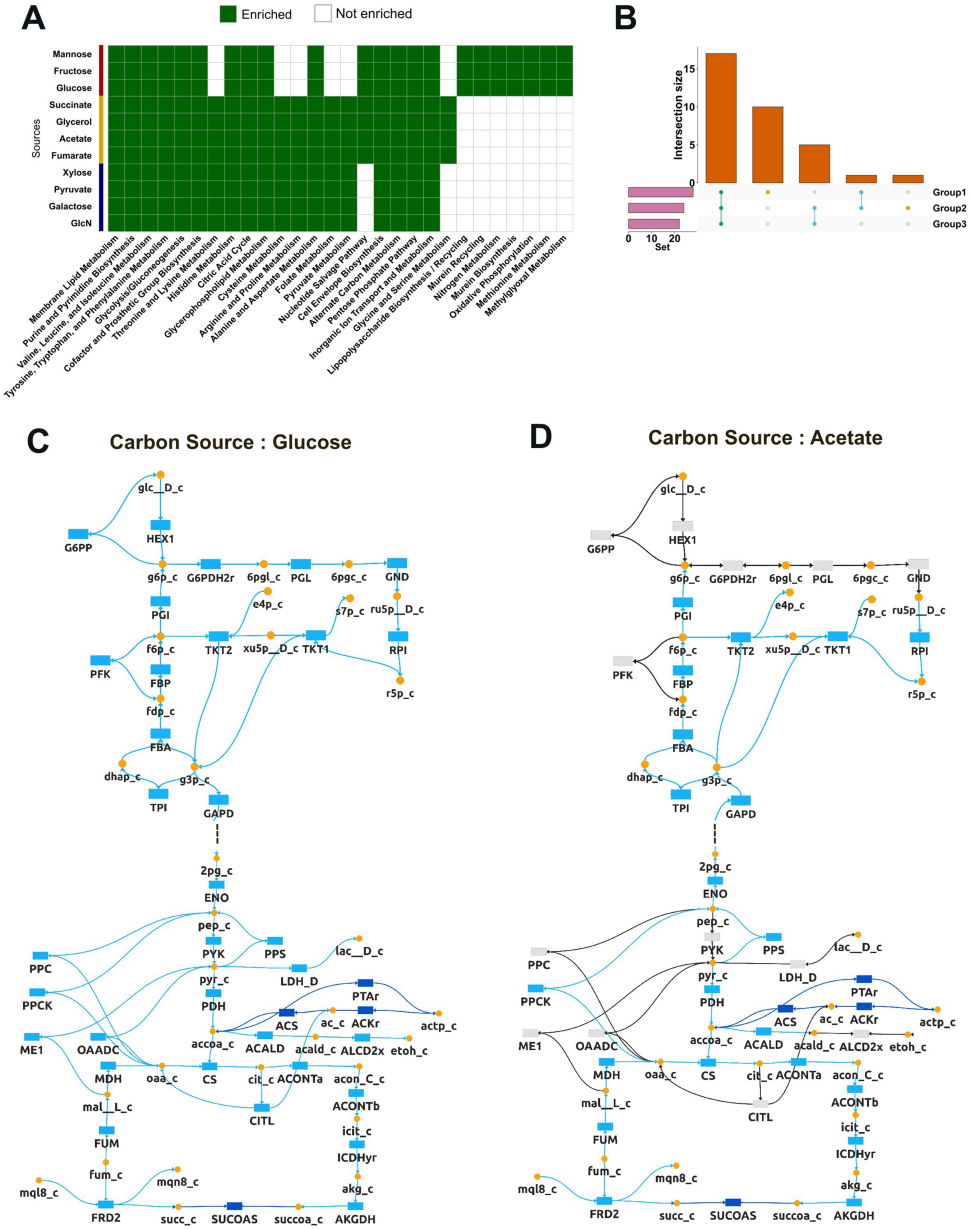
NAViFluX predicts interpretable metabolic adaptations of *Escherichia coli* to carbon sources. (A) Heatmap comparing over-representation analysis (ORA) of flux-active pathways across 11 carbon source conditions. Green indicates enriched pathways, while white denotes non-enriched pathways. Condition-specific flux profiles cluster into three distinct enrichment groups (Groups 1–3). (B) UpSet plot showing the intersection of enriched pathways among the three groups, highlighting pathways common to all groups as well as those uniquely enriched in individual groups. (C) Flux visualization on a merged central carbon metabolism subnetwork for the reference carbon source: glucose (Group 1). Colour gradient corresponds to the average of minimum and maximum FVA flux values. Directions display flow of metabolites through the network. (D) Flux visualization of a merged central carbon metabolism subnetwork for the non-preferred carbon source: acetate (Group 2).

For each carbon source, experimentally detected metabolites entirely formed a subset of flux-active metabolites (Supplementary Figure 1), confirming that the measured metabolites were growth-associated and that inferred fluxes reliably represent nutrient-specific metabolic potential. Condition-specific flux-active reactions were subjected to ORA using the “Functional Enrichment Analysis” module. Comparing enriched processes across the 11 conditions revealed three clusters of enrichment profiles, demonstrated shared catabolic pathway usage (Fig. 3A). Enriched pathways spanned central metabolic, anaplerotic, and biosynthetic processes, with 10 pathways unique to Group 1, one unique to Group 2, and 17 common to all three groups (Fig. 3B, Supplementary File 2).

Using NAViFluX’s flux-oriented visualizations, flux distributions under glucose and acetate uptake were compared on a merged subnetwork combining Glycolysis/Gluconeogenesis, the Citric Acid Cycle (TCA cycle), the Pentose Phosphate Pathway (PPP), and Anaplerotic Reactions. FVA-derived flux weights were overlaid directly onto this subnetwork. Glucose growth produced a high glycolytic flux state with an optimized PPP for biomass synthesis and active overflow metabolism generating lactate, acetate, and ethanol (Fig. 3C). In contrast, acetate growth revealed a strictly gluconeogenic state, with carbon flowing from the TCA cycle into gluconeogenesis for hexose phosphate production via non-oxidative PPP (Fig. 3D). Inactivation of oxidative PPP likely prevents carbon loss as CO_2_, reflecting acetate’s role as a frugal carbon source, while an active TCA cycle with no overflow metabolism indicates acetate is just sufficient to maintain acetyl-CoA and oxaloacetate pools.

Overall, this case study demonstrates NAViFluX’s ability to tune model constraints, perform flux and ORA analyses, and generate mechanistically interpretable, flux-weighted network visualizations.

#### 3.2.2 Case study 2: Improving essential gene predictions in *E. coli* using intuitive single-gene deletion simulations

**Figure 4:**
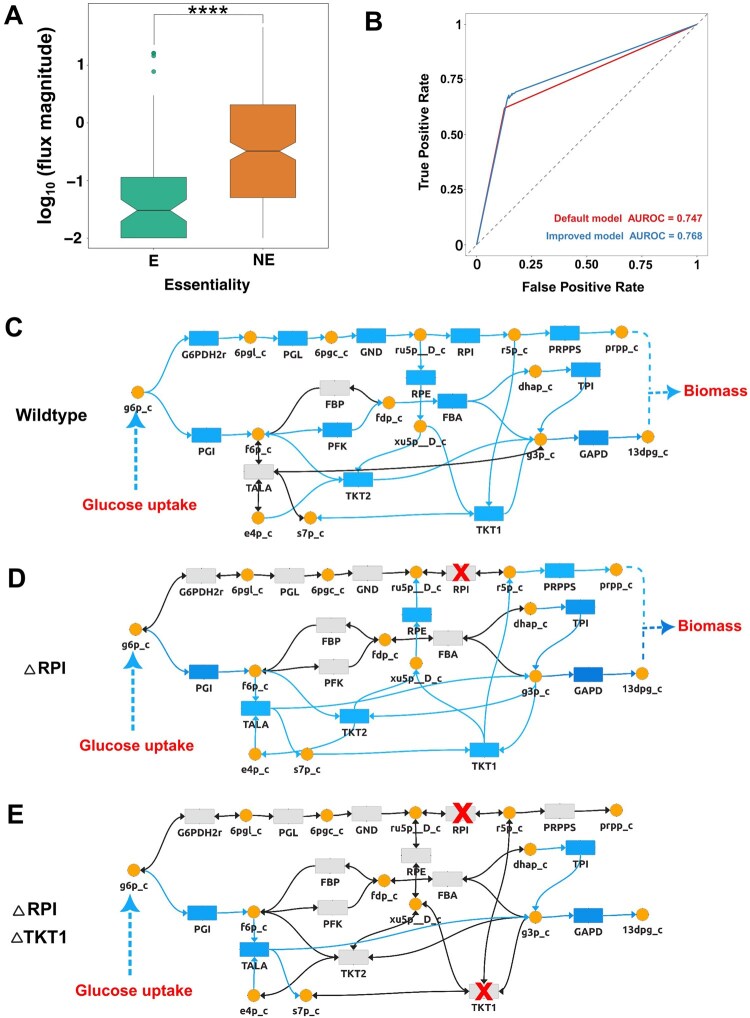
NAViFluX based prediction of metabolic gene/reaction essentiality and mechanistic interpretation through flux visualization. (A) Comparison of reaction fluxes in the wild-type *E. coli* iJO1366 GSMN between reactions associated with the experimentally essential [E] and non-essential [NE] genes as reported in the KEIO single-gene deletion library. Plotting is performed in log-scale of absolute flux. Overall, non-essential reactions exhibit significantly higher fluxes than essential reactions (Wilcoxon test, *P* = 4.36 × 10^−11^). (B) Receiver operating characteristic (ROC) curves comparing essential gene prediction performance using default model constraints (red; auROC = 0.747) and minimally refined internal constraints (blue; auROC = 0.768), demonstrating improved prediction performance upon constraint refinement. Constraint refinement and genome-scale gene knockouts were implemented in NAViFluX. (C) Flux visualization in the *prpp* synthesis subnetwork under wild type conditions. Cycle-free flux balance analysis (cFBA) indicates that *prpp* synthesis is preferred via the oxidative pentose phosphate pathway mediated by ribose-5-phosphate isomerase (RPI). (D) Flux visualization in the *prpp* synthesis subnetwork upon single reaction knockout of RPI in the *E. coli* iJO1366 model. (E) Flux visualization in the *prpp* synthesis subnetwork upon combinatorial knockout of RPI and TKT1.

NAViFluX predicts essential metabolic genes and reveals the mechanisms underlying their essential nature. We evaluated NAViFluX’s gene/reaction perturbation predictions against the KEIO single-gene deletion library for *E. coli* K-12 MG1655, which catalogues the essentiality phenotype of 3,985 nonessential and 303 essential genes (Baba et al. 2006). The iJO1366 GSMN was constrained for growth in MOPS medium and cycle-free FBA (cFBA) was performed using the “Flux Analyses” module. cFBA estimates flux profiles that maximize a user-defined cellular objective while correcting for futile cycles. Notably, non-essential reactions carry significantly larger flux magnitudes than essential reactions (Fig. 4A), reflecting their involvement in flexible, redundant routes while essential reactions act as metabolic “chokepoints” in non-redundant, linear pathways.

Single-gene deletion simulations using NAViFluX yielded an auROC of 0.74 against KEIO essentiality data, reflecting the baseline predictive strength of the iJO1366 model. We hypothesized that prediction accuracy depends on the accuracy of internal network constraints. As an illustrative example, phosphoribosyl pyrophosphate synthetase (PRPPS), an experimentally essential enzyme was incorrectly predicted as non-essential. Visual exploration in NAViFluX revealed that the Ribose-1,5-bisphosphokinase (R15BPK) reaction provides an alternative route to phosphoribosyl pyrophosphate (*prpp*), masking PRPPS essentiality. Since R15BPK is dispensable in *E. coli*, its flux was constrained to zero, rendering PRPPS indispensable. Constraining glucose catabolism equally through periplasmic glucose transport (GLCptspp) and hexokinase (HEX1) improved essentiality prediction to auROC = 0.76 (Fig. 4B), demonstrating NAViFluX’s utility in visually guided, iterative model correction.

To mechanistically trace glucose catabolism towards *prpp* synthesis (PRPPS), subsystems like glycolysis, PPP, and histidine metabolism were merged within NAViFluX. The resulting subnetwork revealed two parallel glucose catabolic routes: RPI-mediated oxidative PPP and TKT1-mediated non-oxidative PPP. Overlaying cFBA flux results confirmed oxidative PPP as the preferred route for *prpp* synthesis under wild-type conditions (Fig. 4C). Single knockout of RPI redirected flux through TKT1-mediated non-oxidative PPP, maintaining *prpp* synthesis (Fig. 4D). However, combinatorial knockout of both RPI and TKT1 eliminated *prpp* production entirely, rendering the double knockout lethal, an observation which intuitively emerges from the merged network visualization (Fig. 4E).

Overall, this case study demonstrates that NAViFluX enables tunable gene essentiality predictions and uncovers visual, mechanistic insights into metabolic redundancies and vulnerabilities.

#### 3.2.3 Case study 3: Engineering an *E. coli* metabolic network for carbon fixation

To illustrate NAViFluX’s utility in simulating engineered metabolism, we reproduced a study where *E. coli* BW25113 was engineered to synthesize sugars solely from CO_2_ via incorporation of Calvin-Benson-Bassham (CBB) cycle enzymes, promoting hemiautotrophic growth (Antonovsky et al. 2016). A “mutant” metabolic network was generated by simulating knockouts of phosphoglycerate mutase (PGM), glucose-6-phosphate dehydrogenase (G6PDH2r), phosphofructokinase (PFK), and glyoxylate shunt reactions (ICL, MALS, ICDHx), alongside addition of non-canonical CBB enzymes RuBisCO and phosphoribulokinase (RBPC and PRUK) retrieved from the integrated BiGG database via NAViFluX’s “Model Editing Options.”

**Figure 5:**
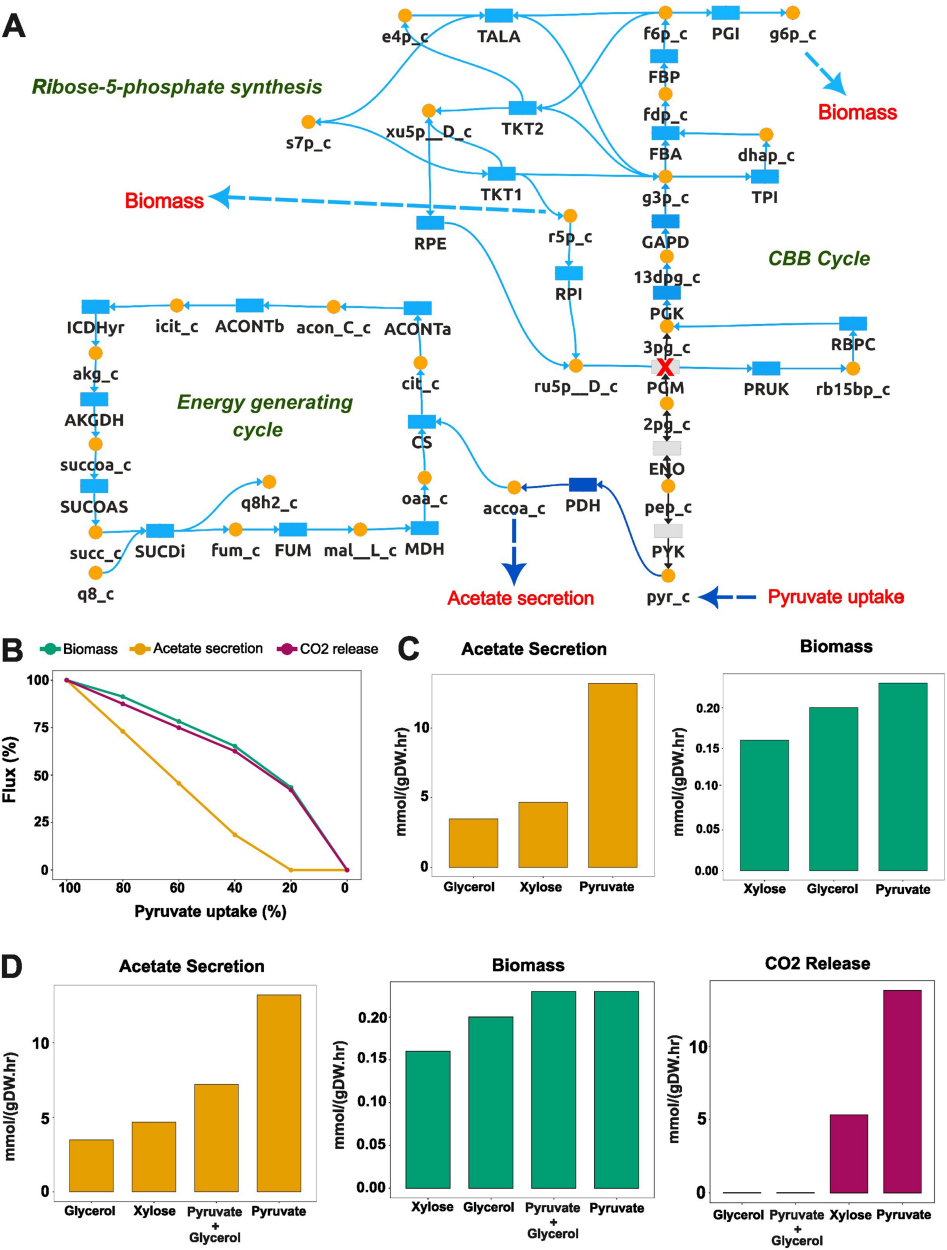
NAViFluX-enabled simulation of hemiautotrophic growth in engineered *E. coli* and its optimization. (A) Flux visualization of pyruvate fate in the engineered *E. coli* iJO1366 model simulated for hemiautotrophic growth. NAViFluX analyses and visualization reveals the emergence of two disjoint but redox-coupled functional flux modules: a CBB module dedicated to CO_2_ fixation and precursor biosynthesis, and an energy generating module fueled by pyruvate oxidation. (B) Effect of progressively reducing pyruvate uptake on biomass formation, acetate secretion, and CO_2_ release. (C) Comparison of acetate secretion and biomass production under individual carbon sources (glycerol, xylose, and pyruvate). (D) Maximization of CO2 fixation into biomass with minimal overflow metabolism.

Constraining pyruvate uptake to 16 mmol/(gDW·hr), cFBA yielded the previously reported growth rate of 23 mmol/(gDW·hr) (Antonovsky et al. 2016). Tracing pathway fluxes confirmed the existence of two disjoint functional modules: a CBB module comprising upper glycolysis and PPP dedicated exclusively to CO_2_ fixation via RBPC and PRUK, enabling regeneration of intermediates and sugar phosphates for biomass synthesis; and an energy module utilizing pyruvate for ATP synthesis and for supplying reducing equivalents (Fig. 5A). This separation of carbon flow between energy generation and CO_2_ fixation recapitulates the reported hemiautotrophic phenotype.

The above constraints led to excessive acetate secretion, indicating carbon loss through overflow metabolism suggesting inefficient carbon fixation. Additionally, a net non-zero CO_2_ release was maintained under optimal pyruvate uptake. To identify conditions minimizing these inefficiencies, pyruvate uptake was progressively reduced in NAViFluX while monitoring CO_2_ and acetate exchange rates. At a pyruvate uptake of 3.2 mmol/(gDW·hr), that is approximately 20% of its optimal rate, acetate secretion reached zero and net CO_2_ release was eliminated, indicating complete CO_2_ utilization (Fig. 5B). However, this came at the cost of reducing the growth rate by 50%.

We hypothesized that a supplementary carbon source could restore growth while maintaining maximum CO_2_ fixation and minimal overflow. Comparing individual carbon sources revealed that glycerol led to the minimal acetate overflow while pyruvate produced maximal biomass (Fig. 5C). Interestingly, a combination of suboptimal pyruvate (4 mmol/gDW/hr) and glycerol (7 mmol/gDW/hr) restored biomass to levels equivalent to default pyruvate uptake while reducing acetate release by 50% (Fig. 5D). Importantly, CO_2_ release under this combination was nearly eliminated mirroring the glycerol-only condition achieving maximal biomass and CO_2_ fixation simultaneously.

This case study demonstrates that NAViFluX not only reproduced the previously reported hemiautotrophic pathway modules but extended the analyses to rationally optimize CO_2_ fixation efficiency showcasing NAViFluX as a powerful platform for designing, simulating, and interpreting engineered metabolic states.

## 4 Discussion and conclusion

NAViFluX provides an integrated, visualization-centric environment that enables entry-level users to perform complex analyses, data-oriented visualizations, and reaction/pathway-level editing of GSMNs. Its core philosophy is to eliminate the traditional separation between mathematical analyses and network visualization allowing users to manipulate constraints, add or delete reactions, merge pathways, and run analyses while immediately inspecting results on pathway subnetworks. NAViFluX thus shifts GSMN usage from script-driven workflows to visually guided, iterative reasoning about metabolic network organization. The key distinguishing features of NAViFluX include native pathway/subsystem map generation with pathway-specific layouts, BiGG/KEGG database informed reaction editing, *de novo* GSMN model building, functional enrichment and topology analyses, and exportable models and maps compatible with external tools.

Case study 1 demonstrates NAViFluX’s ability to visually distinguish glycolytic versus gluconeogenic states under glucose and acetate growth by overlaying flux profiles on merged pathway subsystems. By identifying coupling between pathways, NAViFluX provides mechanistic interpretations for carbon source preference and its consequences on biomass and overflow metabolism, transforming numerical flux distributions into pathway-level, interpretable metabolic views.

Case study 2 highlights that NAViFluX goes beyond passive pathway viewing to actively support iterative model correction. Visually identifying an dispensable bypass reaction (R15BPK) enabled targeted constraint adjustment to recover experimentally validated essential genes (e.g. PRPPS), improving model prediction performance. Similarly, merged subnetworks spanning glycolysis and the pentose phosphate pathway (PPP) clarified the joint control of oxidative and non-oxidative PPP on *prpp* availability, identifying RPI and TKT1 as rational targets for combinatorial perturbations. NAViFluX thus functions as an interactive sandbox where consequences of constraint changes and reaction modifications are immediately accessible at the pathway level.

Case study 3 extends NAViFluX’s utility to engineer metabolic states. By incorporating CBB cycle reactions, constraining native reactions, and simulating combinatorial carbon uptake, NAViFluX reproduced the previously reported hemiautotrophic behavior and further optimized carbon fixation efficiency. This demonstrates the platform’s capacity to design and interpret engineered flux modules and evaluate trade-offs between growth, carbon catabolism, and by-product secretion.

Despite its broad utility, performance for large, merged networks scales with available computational resources. Future developments include support for community and microbiome models, integration with emerging metabolic representations, and compatibility with automated genome-based reconstruction tools (Machado et al. 2018, Fresno et al. 2025, Moretti et al. 2026). We envision NAViFluX becoming a routine tool in experimental and computational laboratories alike for exploratory hypothesis generation and mechanism-oriented communication of metabolic model results.

## Supplementary Material

btag191_Supplementary_Data

## Data Availability

All source code and supplementary files associated with the case studies are publicly available via Zenodo at  https://zenodo.org/records/19107831. In addition, the complete source code, detailed documentation, test datasets, and step-by-step installation instructions are accessible through the project’s GitHub repository at https://github.com/bnsb-lab-iith/NAViFluX.
