# Supplementary figures and images for: NAViFluX: a visualization‑centric platform for interactive analysis, refinement and design of genome‑scale metabolic networks
Source: Bioinformatics. 2026 Apr 20;42(5):btag191. doi: 10.1093/bioinformatics/btag191 (PMC13181184; doi:10.1093/bioinformatics/btag191)

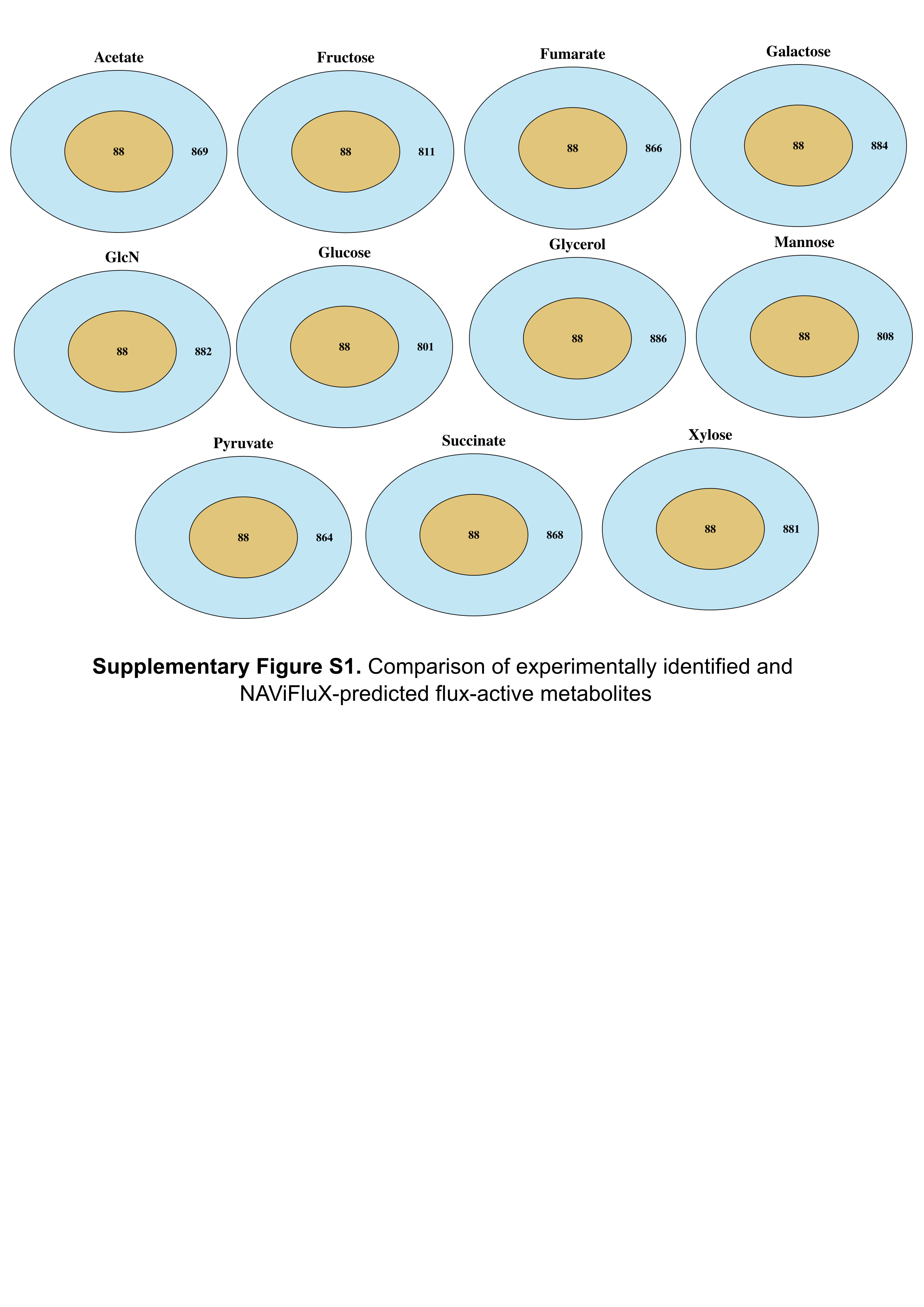

Supplement: btag191_Supplementary_Data [file btag191_supplementary_data.zip › Supplementary Figure 1.tiff]
